# Calibration of the HemoCue point-of-care analyser for determining haemoglobin concentration in a lizard and a fish

**DOI:** 10.1093/conphys/cow006

**Published:** 2016-03-12

**Authors:** Sarah J. Andrewartha, Suzanne L. Munns, Ashley Edwards

**Affiliations:** 1 CSIRO Agriculture, Integrated and Sustainable Aquaculture Production, Castray Esplanade, Hobart, TAS 7000, Australia; 2 Institute of Marine and Antarctic Science, University of Tasmania, Castray Esplanade, Hobart, TAS 7000, Australia; 3 Biomedical Sciences, College of Public Health Medical and Veterinary Sciences, James Cook University, Townsville, QLD 4812, Australia; 4 School of Biological Sciences, University of Tasmania, Private Bag 55, Hobart, TAS 7001, Australia

**Keywords:** Diploid, fish, HemoCue, haemoglobin concentration, reptile, triploid

## Abstract

Haemoglobin concentration ([Hb]) is measured for a wide variety of animal studies. The use of point-of-care devices, such as the HemoCue, is becoming increasingly common because of their portability, relative ease of use and low cost. In this study, we aimed to determine whether the [Hb] of blue-tongued skink (*Tiliqua nigrolutea*) blood can be determined accurately using the HemoCue and whether the HemoCue overestimates the [Hb] of reptile blood in a similar manner to fish blood. Additionally, we aimed to test whether ploidy affected [Hb] determined by the HemoCue using blood from diploid and triploid Atlantic salmon (*Salmo salar*). The HemoCue Hb 201^+^ systematically overestimated [Hb] in both blue-tongued skinks and Atlantic salmon, and there was no difference between calibration equations determined for diploid or triploid salmon. The overestimation was systematic in both species and, as such, [Hb] determined by the HemoCue can be corrected using appropriate calibration equations.

## Abstract (precis)

Hemoglobin concentration is commonly measured by point-of-care systems for many animal studies. The HemoCue Hb 201+ overestimated hemoglobin in blue-tongued skinks and diploid and triploid Atlantic salmon. The overestimation was systematic in both species, and therefore hemoglobin determined by the HemoCue can be corrected using appropriate calibration equations.

## Introduction

Point-of-care (POC) devices, such as the HemoCue, are becoming increasingly common for animal studies because of their relative ease of use and low cost (for review, see Stoot *et al.*, 2014). The portable nature of POC devices enables technically difficult data to be obtained in remote or challenging locations. Although POC devices can be used to show relative changes within the context of a study (e.g. Munns *et al.*, 2012), it is important to be cautious about interpreting the absolute values and comparing between techniques if validation studies have not been conducted.

The HemoCue Hb 201^+^ system (Angelholm, Sweden) is a small, easy-to-use POC device that determines haemoglobin concentration ([Hb]). It is used widely by health-care providers around the world and commonly used in animal research (Srivastava *et al.*, 2014; Table 1 and references within). In the HemoCue cuvettes, sodium deoxycholate lyses the erythrocytes, then sodium nitrite converts the haemoglobin to methaemoglobin, which forms the azidemethaemoglobin complex because of the presence of sodium azide. The HemoCue measures absorbance at two wavelengths (565 and 880 nm), which additionally compensates for turbidity. Importantly, the internal calibrations of the HemoCue system have been developed for human blood, in which the erythrocytes are non-nucleated, and thus measurements of other species may be compromised owing to differences in erythrocyte size and structure.

**Table 1:** Summary of studies in a range of species that have validated the use of HemoCue for determination of haemoglobin concentration ([Hb])

	Species	Model	Comparison with standard techniques	Validation equation	Reference
Mammals	Cats	HemoCue B	Comparable	Not required	Posner *et al.* (2005)
	Rabbits	Not provided	Comparable	Not required	Neufeld *et al.* (2002)
	Pigs	Not provided	Comparable	Not required	Kutter *et al.* (2012)
Birds	Bar-headed geese	HemoCue201^+^	Overestimation	(HemoCue[Hb]) = 1.408 (Drabkin’s’[Hb]) − 0.272	Harter *et al.* (2015)
Fish	Sockeye salmon, Chinook salmon, Pacific bluefin tuna, Chub mackerel	HemoCue201^+^	Overestimation	[Hb] = 0.815 (HemoCue [Hb]) − 2.198 Combined regression	Clark *et al.* (2008)
	Atlantic salmon	HemoCue201^+^	Overestimation	[Hb] = 0.820 (HemoCue [Hb]) − 8.883	Present study
Reptiles	Blue-tongued skink	HemoCue201^+^	Overestimation	[Hb] = 0.885 (HemoCue [Hb]) + 7.498	Present study

Haemoglobin concentration provides an indication of oxygen carrying capacity and thus is a useful bioindicator for a diverse range of studies, including basic physiology, immunology, toxicology and veterinary practice studies (Salice *et al.*, 2009; Nevarez *et al.*, 2011; Krams *et al.*, 2013). As a result, HemoCue systems have been used to determine [Hb] in a wide variety of mammals, fish, reptiles, amphibians and birds (e.g. Colombelli-Négrel and Kleindorfer, 2008; Bazar *et al.*, 2009; McFarland *et al.*, 2012; Norman *et al.*, 2012; Rummer *et al.*, 2013). However, HemoCue systems have only been validated in mammals, birds and fish (Table 1). In birds and fish, the HemoCue overestimates [Hb] and there is some evidence that species-dependent validations may be required (Table 1 and references within).

Mean corpuscular volume (MCV), the presence of a nucleus and incomplete erythrocyte lysis have been proposed as potential reasons why [Hb] of some vertebrate groups is overestimated by the HemoCue (Arnold, 2005; Clark *et al.*, 2008; Gustavsson, 2015). Presumably, incomplete lysis would lead to less [Hb] being converted into azidemethaemoglobin; hence, and underestimation of [Hb] by the HemoCue. If the presence of the nucleus alters the [Hb] determined by the HemoCue, we expect that [Hb] would be overestimated in all animals with nucleated erythrocytes, as has been shown in fish (Clark *et al.*, 2008). If changes in MCV are responsible, then we would expect that the [Hb] of two species with a comparable MCV would be determined to be similar by the HemoCue.

The aims of this study aimed were as follows: (i) to determine whether the [Hb] of reptile blood can be determined accurately using the HemoCue; and (ii) to determine whether differences in MCV or nucleation of the erythrocytes affects the values produced by the HemoCue. To address the second aim, we used commonly farmed diploid and triploid salmon because triploids have larger erythrocytes (MCV) with larger nuclei than their diploid counterparts, and the MCV of diploid salmon is similar to that of reptiles (Sandnes *et al.*, 1988; Benfey, 1999; Cogswell *et al.*, 2002; Moller, 2014). We hypothesized that the presence of the nucleus in fish and reptile erythrocytes would result in an overestimation of [Hb] by the HemoCue in all three animal groups because of the HemoCue’s internal calibrations being developed for non-nucleated human erythrocytes.

## Materials and methods

Blood was collected from eight male blotched blue-tongued skinks (*Tiliqua nigrolutea*), seven triploid and five diploid Atlantic salmon (*Salmo salar*). Skinks were approximately 350–450 g and were maintained in a captive colony at the University of Tasmania. Salmon of ∼1.5 kg were obtained from a commercial aquaculture facility at Dover, Tasmania and maintained at 16°C in a recirculating aquarium facility at CSIRO, Hobart. Animals were fasted for at least 24 h before approximately 0.4–1.0 ml blood was collected from tail arteries or veins in heparinized plastic syringes and stored on ice for <2 h before use. Skinks were restrained by hand during blood sampling, and all samples were collected from the caudal artery using a heperanized 1 ml needle and 25 gauge syringe within 2 min. Salmon were anaesthetised with Aqui-S (Aqui-S, Australia), then sampled in air within 30 s. Experiments were conducted under University of Tasmania animal ethics permits (A0013794 and A0014283).

A dilution series was created from each blood sample to establish a range of [Hb] that was wider than the biological range for [Hb], which is approximately 30–140 g l^−1^ for blue-tongued skinks and other *Tiliqua* spp. and approximately 89–104 g l^−1^ in Atlantic salmon (Sandnes *et al.*, 1988; Moller, 2014; S. Munns, A. Edwards and S. Andrewartha, unpublished data). Each blood sample was divided into five aliquots (including whole blood). Two aliquots were haemodiluted with different volumes of 0.6 and 0.9% saline, which are approximately isotonic with reptile and fish blood, respectively (Thorarensen *et al.*, 1991; Martinez-Jimenez and Hernandez-Divers, 2007). A further two aliquots were haemoconcentrated by centrifuging the sample, removing plasma and subsequently resuspending the erythrocytes in the reduced volume of plasma. Once the dilution series was created, [Hb] was determined using both the HemoCue and the well-established Drabkin’s method (Sari *et al.*, 2001).

### HemoCue

Samples were mixed using a vortex, then 10 µl was loaded into HemoCue cuvettes using a pipette. Skink and salmon blood samples were read 1 and 7 min after being loaded into cuvettes, respectively. This reflected the longer time required for the reactions within the cuvettes to stabilize for salmon blood as has been reported previously (Clark *et al*., 2008). Preliminary experiments measured [Hb] every 30 s for 10 min to determine the appropriate incubation time. Replicate readings were obtained until two concurrent readings varied by <2g l^−1^ (generally, duplicate readings per sample).

### Drabkin’s method

In triplicate, 6 µl of blood was added to 600 µl aliquots of Drabkin’s reagent (D 5941; Sigma Aldrich, Castle Hill, NSW, Australia). Samples were immediately mixed using a vortex and incubated for at least 1 h at room temperature. Degraded proteins were removed by centrifuging samples at 10 000***g*** for 10 min. Next, 350 µl of the supernatant was transferred into 96-well plates alongside pure Drabkin’s reagent as a blank. The absorbance was read on a spectrophotometer at 540 nm (Spectramax 190; Molecular Devices, USA).

The [Hb] (in grams per litre) was calculated according to equation (1):

(1) $$[\mathrm{Hb}]=(A_{540}-A_{\mathrm{Blank}})\times \;\frac{W_{\mathrm{Hb}}\times D_{F}}{C_{E}\times d\times 1000}$$

where $A_{540}$ is the absorbance of the sample at 540 nm, *A*_Blank_ is the absorbance of the blank (i.e. Drabkin’s reagent) at 540 nm, *W*_Hb_ is the molecular mass of human Hb tetramer (=64 458), *D*_F_ is the dilution factor (=101), *C*_E_ is the extinction coefficient for terameric cyanomethaemaglobin at 540 nm (=44), *d* is the light path in centimetres (=1), and 1000 converts milligrams to grams (Dacie and Lewis, 1975; Clark *et al.*, 2008).

In practice, [Hb] (in grams per litre) was calculated according to equation (2), as follows:

(2) $$[\mathrm{Hb}]=(A_{540}-A_{\mathrm{Blank}})\times 148$$

The relationship between [Hb] determined by the HemoCue and Drabkin’s reagent was established using least-squares regression. The slopes of the data from each species were compared with the line of equality using ANCOVA in R-studio version 0.98.1087.

The difference in [Hb] measured by the two methodologies (Δ[Hb]) was determined using equation (3), as follows:

(3) $$\Delta [\mathrm{Hb}]=100\times \frac{\begin{array}{l} ([\mathrm{Hb}]\;\mathrm{determined}\;\mathrm{by}\;\mathrm{HemoCue}\\ \;\;-[Hb]\text{ determined by Drabkin's)} \end{array}}{[\mathrm{Hb}]\text{ determined by Drabkin's}}$$

## Results and discussion

**Figure 1:**
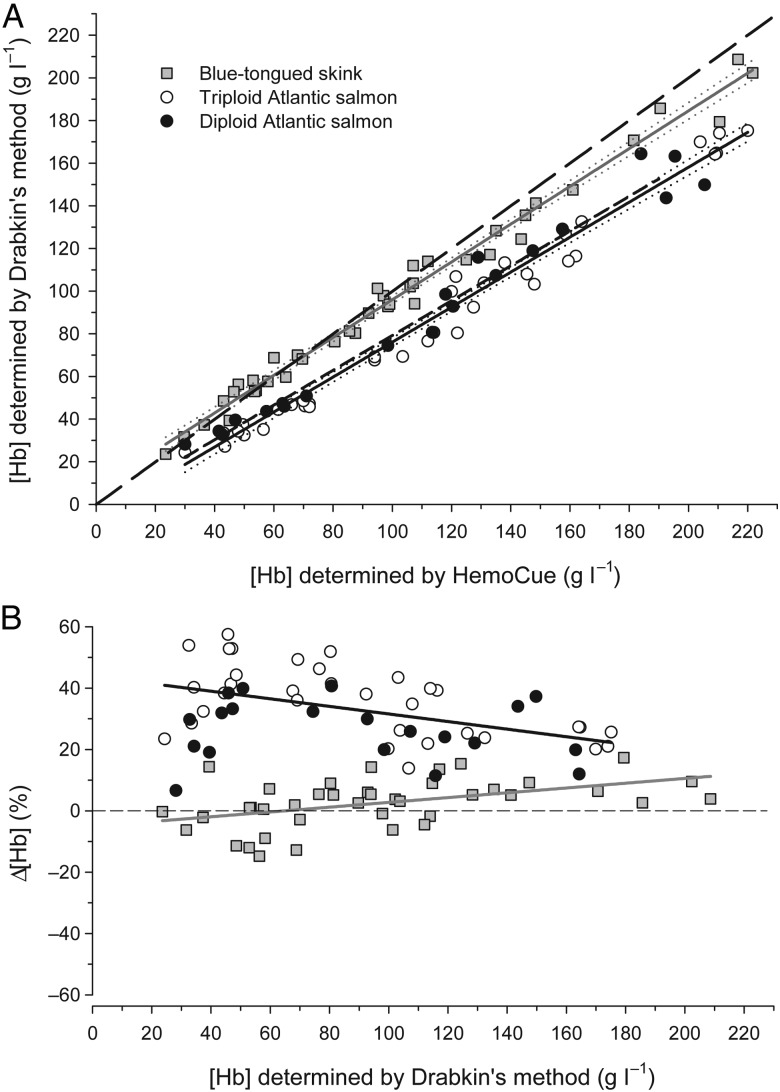
(**A**) Comparison of haemoglobin concentration ([Hb]) determined using HemoCue system and Drabkin’s method in blue-tongued skinks (grey squares), triploid (open circles) and diploid (filled circles) Atlantic salmon. Continuous lines are species-specific regressions for skinks (*y* = 0.885*x* + 7.498, *r*^2^ = 0.985) and salmon (*y* = 0.820*x* − 5.831, *r*^2^ = 0.975) with 95% confidence intervals (dotted). The long dashed line represents the line of equality and the short dashed line is fish blood taken from Clark *et al*. (2008). (**B**) [Hb] measured using Drabkin’s method against the difference in [Hb] determined by Drabkin’s method and the Hemocue {Δ[Hb], equation (3)}. The dashed line is the line of equality.

The [Hb] determined by the HemoCue Hb 201^+^ was overestimated in both Atlantic salmon and blue-tongued skinks (Fig. 1). In both species, the systematic nature of the HemoCue overestimation of [Hb] meant that calibration equations to correct data collected by this rapid POC system can be used reliably. Systematic overestimation of [Hb] by the HemoCue has previously been reported in fish and birds (Clark *et al.*, 2008; Harter *et al.*, 2015).

The slopes of the relationships between [Hb] determined by the HemoCue and Drabkin’s method were not significantly different between diploid and triploid Atlantic salmon (*P* > 0.05); therefore, the data were pooled for further analysis (Fig. 1). Equation (4), which describes the relationship between the [Hb] values for Atlantic salmon determined by the two methods, has a slope that is significantly different from the line of equality (*P* < 0.010), as follows:

(4) $$\begin{array}{l} \mathrm{Drabki}n^{\prime }s\;[Hb]=0.820\;(HemoCue\;[Hb])-5.831,\\ \;\;\;\;\;\;\;\;\;\;\;\;\;\;\;\;\;\;\;\;\;\;\;\;\;\;\;\;\;\;\;\;\;\;\;\;\;\;\;\;r^{2}=0.977 \end{array}$$

The slope and elevation of equation (4) were not significantly different from those of the validation equation determined for four fish species, including two salmonids, by Clark *et al*. (2008; *P* < 0.001; Table 1). The similarities suggest that a common calibration can be used for salmonids and, potentially, other saltwater fish.

Likewise, the [Hb] of blue-tongued skinks was overestimated by the HemoCue (Fig. 1). Equation (5), which describes the relationship between the [Hb] values determined by the two methods, has a slope that is significantly different from the line of equality (*P* < 0.010), as follows:

(5) $$\mathrm{Drabki}n^{\prime }s\;[Hb]=0.885\;(HemoCue\;[Hb])+7.498,\;r^{2}=0.993$$

The overestimation of [Hb] predominantly occurred at the upper end and at [Hb] values beyond the biological range of approximately 30–140 g l^−1^ for blue-tongued skinks and other *Tiliqua* spp. (Moller, 2014; S. Munns, A. Edwards and S. Andrewartha, unpublished). The consequence for an uncalibrated [Hb] measurement of 140 g l^−1^ at the upper end of the physiological range would be an overestimation of ∼7% (131 g l^−1^; Fig. 1B). Although this is smaller than the 30% overestimation that would occur in fish at the same [Hb], it is not inconsequential, and best practice would correct all blue-tongued skink [Hb] measurements that are determined by the HemoCue.

The presence of the nucleus, the larger size of the erythrocytes (i.e. mean corpuscular volume) and incomplete red blood cell lysis have been proposed as potential causative factors and will be discussed in relationship to the data collected in the present study (Arnold, 2005; Clark *et al.*, 2008; Gustavsson, 2015). No differences were observed between triploid and diploid salmon [Hb] calibrations in spite of triploid salmon having an MCV that is ∼40% greater than diploids (see Benfey, 1999, and references within). This is in contrast to the larger inaccuracies measured in ostrich erythrocytes manipulated to have larger MCV (Gustavsson, 2015). Likewise, if the overestimation was the result of differences in MCV we would expect [Hb] to be overestimated to a similar degree in skinks and Atlantic salmon because they have similar MCV. Mean corpuscular volume is 441–553 × 10^−15^ l in Atlantic salmon and 422 × 10^−15^ l in *Tiliqua* spp. (Sandnes *et al.*, 1988; Moller, 2014). Instead, the overestimation was lower in skinks compared with Atlantic salmon.

If the presence of the nucleus is altering the readings of [Hb] in blood from animals with nucleated compared with non-nucleated erythrocytes, then we would expect that a larger volume of nuclear material would magnify the overestimation of [Hb]. The volume of the nucleus is ∼1.7 times larger in triploid (78 µm^3^) compared with diploid (45 µm^3^) salmon; however, there are 1.6 times more erythrocytes in diploid blood (1.08 cf. 0.68 million mm^−1^; Benfey and Sutterlin, 1984). These two opposing factors are likely to result in the same amount of nuclear material in both ploidies; hence, we would not expect calibration differences if the nucleus was the cause of [Hb] overestimation. In general, the size of reptile erythrocyte nuclei is similar to those of fish (Hartman and Lessler, 1964). Thus, the larger overestimation of [Hb] in salmon compared with skinks suggests that a factor other than erythrocyte nucleation is responsible for the overestimation. This finding, that the presence of the nucleus is not causing the overestimation in [Hb], may be relevant for measuring [Hb] on fish and reptile blood using other POC devices, although validation for the other POC devices and species would be required. Owing to a lack of data on the species tested in the present study (blue-tongued skinks), we cannot completely rule out erythrocyte nuclei size differences between the two species in this study and possible influences on [Hb] determination.

Potentially, the structure and/or composition of the Hb itself differs between groups and produces the variation in [Hb] measured by the HemoCue. The haemoglobin of adult humans is composed predominantly (97–98%) of subtype Hb_a_, with Hb_a2_ making up a further 2% and Hb_F_ ∼1% (Maniatis *et al.*, 1980). Fish commonly have a larger number of haemoglobin subtypes (e.g. 8–14 in salmonids), which are thought to facilitate oxygen delivery over their wider operational body temperature range (Weber, 2000). It is possible that the structure or incomplete conversion of one or more of these subtypes to azidemethaemoglobin (HemoCue) or cyanmethaemoglobin (Drabkin’s method) results in different [Hb] determined by the two methodologies. We were unable to find data in the literature that examines whether Hb subtypes have different absorbance properties; thus, this remains an avenue for further study.

Although it cannot be ruled out, incomplete lysis of erythrocytes is also unlikely to be causing the overestimation of [Hb] because it would result in less azidemethaemoglobin and a consequent underestimation of [Hb] measured by the HemoCue. Regardless, the uncertainty surrounding why [Hb] is overestimated in vertebrates with nucleated erythrocytes should not deter use of the HemoCue after appropriate validation. The mechanisms behind the overestimation of [Hb] by the HemoCue are an avenue for further research.

In summary, although the HemoCue 201^+^ system overestimated [Hb] in both blue-tongued skinks and Atlantic salmon, the error was systematic and can be corrected using appropriate calibration equations. Owing to some interindividual differences (i.e. variance), care should be taken when interpreting [Hb] calculated for individual animals. However, best practice for determining salmonid [Hb] using the HemoCue would be to correct [Hb] values using equation (4), which is appropriate for both diploid and triploid Atlantic salmon and, potentially, for other salmonid species, and use mean values for several animals. Although the overestimation was considerably lower, equation (5) should be used for best practice in blue-tongued skinks, and we would encourage appropriate validation before using the HemoCue to determine [Hb] in other reptiles.

## Funding

This study was funded by the R. Lobb endowment to S.L.M. and by the Commonwealth Scientific and Industrial Research Organisation (CSIRO) and Salmon enterprises of Tasmania Pty Ltd funding to S.J.A.
